# Delusions of a Magic Man Shared by Codependent Twin Sisters

**DOI:** 10.1155/2022/9126521

**Published:** 2022-05-13

**Authors:** Anam Shaikh, Randy Lai

**Affiliations:** Department of Psychiatry, St. John's Episcopal Hospital, 327 Beach 19th street, Far Rockaway, NY 11691, USA

## Abstract

Folie à deux has been called double insanity or shared psychotic disorder. In this report, we describe the case of twin sisters who presented with identical delusions. Both sisters reported a “magic man” lived in their bodies and made them weak. Both underwent treatment with similar medications: the primary case with risperidone and the secondary case with paliperidone. The delusions of the primary case slowly abated with antipsychotics, whereas those of the secondary case completely resolved within a short duration after admission when the twins were separated in different hospital units.

## 1. Introduction

Here, we have an interesting case of identical twin sisters presenting simultaneously with similar delusions, also known as “folie à deux.” This case is unique where both patients not only have psychiatric, but also medical complaints that stumped the treatment team due to the elusive nature of the diagnosis of folie a deux. The criteria for diagnosis of “folie à deux” and its nomenclature have changed over the years. In 1877, two French psychiatrists, named Lasegue and Falret, coined the term “folie a deux” [1]. Based on the current DSM-5 diagnostic criteria, “folie à deux” falls under *other specified schizophrenia spectrum and other psychotic disorders* with the specifier *delusional symptoms in partners of individuals with delusional disorder* [2]. Individuals with this disorder must not meet the criteria for schizophrenia and must have impaired social functions.

The disorder is characterized by shared delusions between individuals who typically live together, are socially isolated, and are close family members [1]. The individual who first develops the delusion is usually referred to as the “primary case,” who is often chronically ill and is the influential partner. The “secondary case” is usually less intelligent and more gullible or passive than the primary case [3]. The incidence and prevalence figures are unknown, and the condition is believed to be underdiagnosed [4].

## 2. Case Presentation

62-year-old, single, unemployed twin sisters simultaneously visited the emergency department of our hospital for multiple somatic complaints caused by a “magic spirit.”

### 2.1. Primary Case

The primary case lived with her twin sister in a private apartment. She had a psychiatric history of schizophrenia, diagnosed at the age of 18 years, and a medical history of asthma, cholelithiasis, and diverticulosis. She presented to the emergency room with a chief complaint of weakness abdominal pain and stated “the spirits raped” her causing the pain for the last two weeks. She reported that her pain gets better when the spirits are done having intercourse with her. The patient shared that she and her twin sister had black magic performed on them by the same magician in their home country and stated that this “magician” controlled her body, lived in her body, made her feel weak, and always followed her around. Physical exams did not reveal any significant abnormalities. Laboratory results including complete blood count (CBC), comprehensive metabolic panel (CMP), thyroid function tests, and head computed tomography (CT) were normal. The abdominal CT without contrast revealed a nonexistent gall bladder due to a history of cholecystectomy. The primary case was treated with oral risperidone, and over the course of seven days, her medication was titrated up to risperidone 2 mg daily. Her delusions improved gradually as per repeated clinical global impression severity (CGI-s) and clinical global impression improvement (CGI-i) scales [5].

### 2.2. Secondary Case

**Figure 1:**
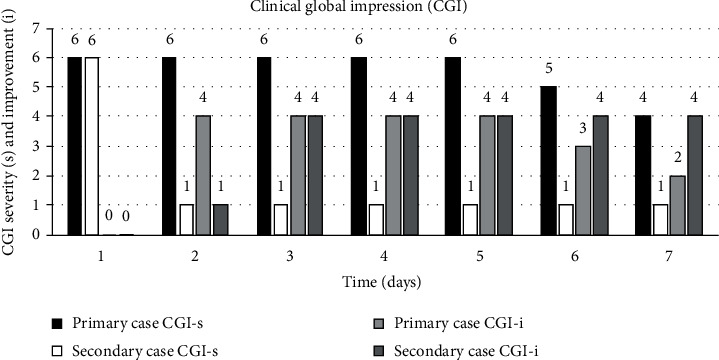
On the day of the hospital presentation, both the primary and the secondary case's CGI-s were scored at 6. After separation to different units, the primary case did not show much improvement as indicated by the CGI-i, but the secondary case showed significant improvement.

The secondary case had a history of unspecified psychosis, diagnosed at the age of 52 years, and a medical history of hypothyroidism. She presented simultaneously to the emergency room with her twin sister with the chief complaint of feeling sick and stated “because a man lives in my body and in my house.” She believed that the man had been controlling her body and mind since childhood and would make her feel “sick and weak.” She called this man “magic man” and “black magic spirit nightmare” who would also rape her at night and follow her everywhere. She reported that her twin sister also shared these symptoms. Physical exam did not reveal any significant abnormalities. CBC, CMP, thyroid function tests, and head and abdominal CT were all normal. The secondary case was treated with oral paliperidone 1.5 mg daily on the second day of admission and was given paliperidone injection 156 mg on the 6th day of her hospitalization. Her delusions improved significantly on the second day after being admitted to another psychiatric unit separated from her sister (see Figure 1).

The two patients' psychiatric and social histories were relatively similar, except that the primary case was diagnosed with schizophrenia at the age of 18 years, while the secondary case began to have psychosis at the age of 52 years. The twins' family members mentioned that the delusions of the secondary case intensified when she was around the primary case and that she denied the occurrence of any delusions when separated from the primary case. There was no known family history of psychiatric disorders with the exception of the twins. Both patients had multiple histories of presenting to the emergency department on the same day with similar chief complaints. They were often hospitalized in the medical units for further workup of their complaints, but laboratory and imaging workup usually yielded negative results. They were discharged with psychiatric outpatient follow-up, but they were poorly adherent to treatment.

## 3. Discussion

Historically, this condition is further classified into four subtypes: *Folie imposée*, *Folie simultanée*, *Folie communiquée*, and *Folie induite* [1].

- *Folie imposée*: The delusions of a person with psychosis are transferred to a person who is mentally sound. Both persons are intimately associated, and delusions of the recipient disappear after separation [6]
- *Folie simultanée*: Simultaneous appearance of identical psychosis occurs in two individuals who are both intimately associated and morbidly predisposed [6]
- *Folie communiquée*: The recipient develops psychosis after a long period of resistance and maintains the symptoms even after separation [6]
- *Folie induite*: New delusions are adopted by an individual with psychosis who is under the influence of another individual with psychosis [6]

As per the historical classifications, we believe our case would fit under the description of *Folie imposée* due to the fact the delusions of the secondary case subsided soon after separation. Despite the secondary case being diagnosed with schizophrenia for the past 10 years, there were trends noted in all the previous documentations that the secondary case had resolution of symptoms shortly after they were separated, while the primary case persists to have psychotic symptoms.

Further psychosocial discussion regarding both patients revealed the twin sisters had been living together since birth. Ever since the death of their main support, their mother, when both patients were 50 years of age, this made them more codependent on each other. The twin sisters withdrew from social interactions and became more isolated.

The secondary case appeared to be less independent than the primary case. Both patients would often present to the emergency department on the same day with similar chief complaints. Even though the primary case was known to have medical conditions described earlier, the secondary case also presented with similar concerns, but repeated workup yielded negative results.

After separation into separate units, the secondary case's source of delusional content was no longer present, and the delusion disappeared on the second day of hospitalization.

Knowing the fact that the secondary case's delusion can reignite by simply being in close proximity with the inducer, this complicates outpatient treatment and care as in our case where both patients are biological twins who live together. Given that delusions are fixed false beliefs, it is difficult to treat with pharmacological agents alone. We urge that approaching treatment from a different perspective, such as addressing their living situation of being in close proximity as it is a major factor for their delusion.

More recent findings reveal that females and males are equally affected. Furthermore, the secondary case can be either younger or older than the primary case. The incidence of this condition is similar between married couples and siblings, and the most influential factor is cohabitation. The secondary case may experience hallucinations where the primary did not. There are poor correlations between the secondary case having a lower intelligence than the primary case. Separation is considered as a form of treatment for this condition; however, if separation by itself may not be sufficient, adjunctive treatment with antipsychotics may be required [3, 4].

## 4. Limitations

This is a case report; the literary resources used contained previously accepted diagnostic criteria for “folie à deux.”

## 5. Conclusion

As seen in this case report, the primary case's symptoms revealed slow improvement in conjunction with antipsychotic treatment. In contrast, the secondary case's symptoms improved significantly upon separation in conjunction with antipsychotic treatment. Current treatment recommendations include separation as well as treatment with antipsychotics. The prevalence and incidence of this condition remain unknown, and it is an underdiagnosed condition due to the isolation of most people affected. As this disorder is rare and can be misdiagnosed with other psychiatric disorders and early recognition, and appropriate treatment is crucial.

## References

[1] Al Saif F., Al Khalili Y. (2020). Shared Psychotic Disorder. *StatPearls*.

[2] American Psychiatric Association (2013). *Diagnostic and Statistical Manual of Mental Disorders*.

[3] Arnone D., Patel A., Tan G. M. (2006). The nosological significance of Folie à Deux: a review of the literature. *Annals of General Psychiatry*.

[4] Sharon I. M. (2019). *Shared psychotic disorder: background and criteria, history, subtypes and characteristics*.

[5] Guy W. (1976). *ECDEU Assessment Manual for Psychopharmacology*.

[6] Dewhurst K., Todd J. (1956). The psychosis of association—Folie à deux. *The Journal of Nervous and Mental Disease*.

